# Attitudes towards Human Papilloma Virus Vaccination in the Latin American Andean Region

**DOI:** 10.3390/healthcare5030055

**Published:** 2017-09-08

**Authors:** Oroma Nwanodi

**Affiliations:** Obstetrics and Gynecology Locum Tenens, Salinas, CA 93902, USA; o.nwanodi@juno.com; Tel.: +1-314-304-2946

**Keywords:** Bolivia, cervical cancer, Chile, Colombia, disease prevention, Ecuador, external genital warts, human papilloma virus vaccine, Peru, sexual health

## Abstract

This commentary explores the distribution of human papilloma virus (HPV) and HPV-related diseases, and factors affecting attitudes towards HPV, HPV-related diseases, and HPV vaccination in the Latin American Andean region. Lack of knowledge of HPV, known negative attitudes or incorrect assumptions about HPV, HPV-related diseases, and HPV vaccination provide a basis upon which to develop targeted HPV awareness and preventive health media campaigns. For maximal effect, media campaigns should use the internet, radio, and television to address health care providers, parents, and students. Additional programming can be developed for clinics to use in-house with their clients. Ministries of Education, Finance, and Health all have roles to play to increase national HPV, HPV-related diseases, and HPV vaccination awareness.

## 1. Introduction

Bolivia, Chile, Colombia, Ecuador, and Peru form the Latin American Andean region. In the Andean region, as shown in Table 1, Bolivia and Ecuador have the highest standardized cervical cancer (CC) mortality rates per 100,000 women years of 21.0 and 14.0 [1]. Chile has the lowest adjusted CC mortality rate of 6.0 for the Andean region, and the greater Caribbean, Central and South America region (LAC), except Puerto Rico [1,2]. Consistent with this, in Bolivia and Ecuador, CC is the leading cause of female cancer deaths [1]. Since 2006, prophylactic primary prevention against the 70–75% of CC due to high risk (HR) HPV-16 and -18 has been available from the heat-labile bivalent (2vHPV) and quadrivalent (4vHPV) vaccinations [3,4,5]. The 96.5% efficacious nonavalent (9vHPV) vaccine affords additional primary prevention from CC due to HR HPV-31, -33, -45, -52, -58 [6]. 9vHPV may increase CC prevention to 90% [6].

HPV-16 (82%) and -18 (18%) occur in 18.9% of squamous cell head and neck cancer (HNSCC) in Colombia [7]. Overall, up to 53.6% of oropharyngeal cancer in South America may be HPV attributable [8]. In Latin America HPV is prevalent in up to 36.5% of penile cancer, which is consistent with 36% globally [9,10]. Of these, HPV-16 (68.7%) and -18 (1.5%) occurred in 70.2% of HPV positive penile cancers [9].

HPV-6 and -11 are causative of external genital warts (EGW) and recurrent respiratory papillomatosis (RRP) [11,12,13]. EGW bear all the negative adverse psychological outcomes of sexually transmitted infections (STI) [11]. Treatment of recurrent EGW has significant human and financial costs in Central and South America [14]. In addition to preventing EGW and RRP, it is biologically plausible that 2vHPV, 4vHPV, and 9vHPV would protect against some HNSCC [7,12]. The average female Latin American and Caribbean HPV vaccination coverage is 19% [15]. Low HPV vaccination rates mean that CC mortality, and the incidence of anal, cervical, oropharyngeal, penile, vaginal, and vulvar cancers, EGW, HNSCC, and RRP will not decline as rapidly as possible.

CC mortality is correlated with human papilloma virus (HPV) prevalence and socioeconomic indicators [2]. Despite proven efficacy and calculated cost-effectiveness, the acceptance and cost of HPV vaccination contributes to low vaccination rates [16,17,18,19,20]. Provision of affordable HPV vaccination is the topic of a separate paper. Acceptability of HPV vaccination hinges on beliefs regarding vaccination in general, attitudes towards sexuality and STIs, and attitudes towards cancer prevention [20,21]. Vaccination non-acceptability precludes immunization irrespective of cost.

## 2. Materials and Methods

**Figure 1:**
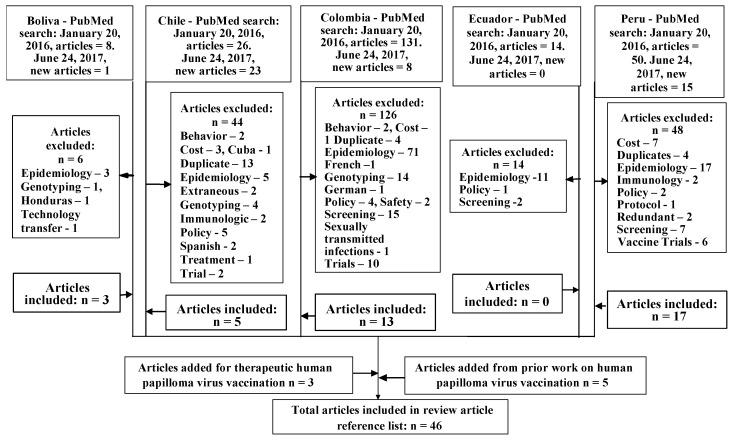
Article selection flow chart.

The PubMed database was searched in January 2016 and June 2017, using the Andean nations Bolivia, Chile, Colombia South America, Ecuador, or Peru, followed by the terms “HPV”, “HPV vaccine”, “HPV vaccination”, “HPV attitudes”, HPV vaccine attitudes”, and “HPV vaccination attitudes”, selecting for human subjects, yielding 276 articles, as shown in Figure 1. Of these, 238 articles were excluded. The most exclusions were for extraneous epidemiology (107 articles), CC screening focus (24 articles), duplication (21 articles), HPV genotyping (19 articles), and clinical trials that did not delve into participants attitudes about HPV and HPV vaccination, or did not include the nations of focus (18 articles). All eliminated articles are indicated in Figure 1. To fill content gaps five articles from prior searches, and three articles on therapeutic CC vaccines were added. The resultant 45 articles incorporated in this review article are summarized in Table 2.

## 3. Results

### 3.1. Population Specific Prevalence and Sequela of HPV in the Andean Region

The global background HPV-16 and -18 prevalence is 32% [13]. Central and South American nations experience dual peak cervical HPV incidence and prevalence in young women and perimenopausal women [19,22]. A dual peak head and neck HPV prevalence has also been found in Colombian men and women with HNSCC [7]. Rural, lower socioeconomic status female Colombian teenagers may have a 35.4 to 36% HPV prevalence [23,24]. For female Colombian teenagers four years after coitarche HPV prevalence increases to 42.5%, whereas other female Colombians have a HPV prevalence ranging from 14.8 to 28.2% [14,23]. Simultaneous multiple HR genotype HPV infections occur in 29.4% of Colombian women [24]. Meanwhile, HPV-6 and 11 are associated with up to 10% of normal or low-grade squamous intraepithelial lesions Pap tests in Colombia [12]. RRP is almost twice as prevalent in Colombian males (60.8%) than females (36.07%) [12].

Almost one-sixth of Bolivian women’s deaths are due to CC [25]. La Paz, Oruro, and Potosi, Bolivia have had CC incidence as high as 53.1, 60.9, and 93.5/100,000, respectively [25]. However, in rural Amazonian Bolivia HPV prevalence ranges from 0 to 16.6% [25]. Peruvian women have a background HPV prevalence of 17.7% versus a global rate of 10% [16]. At 32.7 per 100,000 women, Peru’s CC age-standardized incidence (ASI) is twice that of low- and middle-income countries (LMIC) overall (17.7), and higher than most South American countries (Table 1) [1,4,5,35]. The increased Peruvian CC ASI is attributable to low cytology cover at 51.30, due to a 40.3% Papanicolaou (Pap) test rate, 42.5% sensitivity for cervical intraepithelial neoplasia grade 3 (CIN3), and the 1.7-fold higher Peruvian background HPV rate [2,35].

Mean age of CC occurrence in Colombia is 51.5 years [22]. Irrespective of perceived low- or high-risk for infectious disease, HPV-16 and HPV-18 account for about 63% of CC throughout Colombia [14,22]. Multiple HPV type infections that include HPV-18 form 47% of HPV infections in Colombia, and 56% in Peru, which are higher than worldwide incidence at 20% [22]. From 2001 to 2006 prevalence of HPV-16 and -18 increased from 2.6% to 6.1% in a cohort of 576 low socio-economic status Chilean females ranging from 17- to older than 70-years-old [27]. Consistent with this, from 2012 to 2016 in seven centers in Santiago, dependent upon population subgroup, the combined prevalence of HPV-16 and HPV-18 in cervical cytology ranged from 3.2% to 7.6% [28,29].

Coitarche is a known co-factor for HPV infection [30]. In Peru, median coitarche is 18-years-old [19]. Colombian females’ mean coitarche is 17.1-years-old, and mean menarche to coitarche interval is 4.6 years, with a mean menarche of 12.5-years-old [30]. Finnish females’ mean coitarche is 15-years-old, mean menarche to coitarche interval is 2.6 years, and menarche is 12.4 years-old. Despite almost double the mean menarche to coitarche interval than Finnish females, Colombian females are more likely to have exposure to HPV-6, -11, -16, or -18 (22.3%) and to have abnormal Pap tests (11.6%) than are Finnish females (10.7% and 9.8% respectively) [30]. Consistent with this, Colombian females had a statistically significant greater prevalence of high-risk HPV (35.1%) than Finnish females (22.5%), *p* < 0.001 [30]. Furthermore, female Colombian immigrants to Spain have a HR HPV prevalence of 27% versus 8% in comparable Spanish females [14].

Increased number of sexual partners is another known co-factor for HPV infection [12,13,23,30]. Overall HPV prevalence in Peruvian female sex workers (FSW) ranges from 50.6 to 66.8%, which is comparable to Burkina Faso (66.1%), Guatemala (67.3%), the Philippines (57.2%), and Japan (52.6%) [13]. Peruvian FSW have an oral HPV prevalence of 7.6%, which is consistent with Spanish FSW [26].

### 3.2. Chilean Attitudes towards HPV Vaccination

Chilean adolescents are aware that multiple sexual partners are a CC risk factor (70.8%) and an HPV infection risk factor (78.3%). While 68.2% are aware that unprotected sex facilitates HPV transmission, only 31.1% used condoms [31]. Dependent on household income, parents in the Maule Region will spend an average of US$252.71, and parents in Santiago would pay a mean of US$758 on HPV vaccination for their adolescent daughters. However, the more daughters in the household, the larger the household size overall, the less the amount available per daughter [17,18].

### 3.3. Colombian Attitudes towards HPV, HPV-Related Diseases, and HPV Vaccination

Prior to school-based HPV vaccination in Colombia in 2012, only 44% of Colombian women aged 18- to 69-years-old were aware of HPV, and only 25% of these women were aware of HPV vaccination [11]. Knowledge of CC screening via Pap testing (97.6% of sampled women) did not translate into HPV knowledge [32]. In Arauca, Bogotá, Cartagena, and Manizales parents of daughters younger than 12 years old were resistant to HPV vaccination to prevent STI, and were concerned of sexual disinhibition, but would accept HPV vaccination to prevent CC [38]. Parents of higher socioeconomic status were less accepting of HPV vaccination [38].

Greater HPV and HPV vaccine awareness are found in Colombians who are university educated (65.6–69%, 59%), married (58.5%, 45%), or belong to a health maintenance organization (HMO) (53.5%, 44.9%) [11]. Middle or high socioeconomic class Colombian women are more likely to have a high knowledge of HPV than were those of low socioeconomic class (*p* = 0.002) [32]. The effect of high school or higher education on HPV knowledge is significant (*p* = 0.006) [32]. Colombians are more likely to learn about HPV and the HPV vaccine from social contacts (72.4%, 44.8%), educational establishments (77.8%, 50%), and the media (82.25%, 59.8%), than from health personnel (53.6%, 42.9%) [11].

Colombian women give illness (83%) and CC prevention (28.9%) as reasons why they received HPV vaccination [20]. HPV vaccination was declined for lack of information (30%), impaired access (28.9%), unknown reasons (21%), and neglect (20%). While age, educational level, coitarche, number of partners, and alcohol or drug use are similar, vaccinated Colombian women are more adherent to preventive lifestyles: consistent condom use, use of other family planning methods, and CC screening, than are non-vaccinated Colombian women [20].

EGW more negatively affect sexuality and self-esteem of Colombian women than men (77%, 46%, *p* < 0.001; 90%, 62%, *p* < 0.01) [11]. Colombian men experience decreased adverse effects of EGW with increased age and HPV awareness [11]. Colombian women experience more adverse effects of EGW with increased education (correlating with increased age and socio-economic status), and a more prominent location of EGW on the vulva instead of within the vagina [11].

Columbia has decentralized health care, such that local districts or municipalities independently decide which vaccinations to provide [39]. There are differences in local health administrators’ opinions associated with municipality developmental status. Maternal mortality, teenage pregnancy, and EGW may be perceived as a greater public health concern than CC [39]. Thus, HPV vaccination is perceived as a means to control one of many STIs, but not as the best primary prevention of CC. When local politicians show an interest in the HPV vaccination, a local implementation program is evaluated, instead of waiting for a national HPV vaccination program [39]. Only those local health administrators actively involved in an immunization program may be concerned about the efficacy and safety of the HPV vaccine. Health care administrators believe that their budgets can accommodate either the existing screening for CC or administering the HPV vaccine but not both, therefore, health care administrators may be reluctant to pursue HPV vaccination implementation programs in a timely fashion irrespective of cost-effectiveness analysis availability [39]. One hope is that in the delay HPV vaccine cost will be reduced [39]. Another strategy is to use limited resources to vaccinate FSW or their daughters who are perceived to be most likely to transmit HPV [39].

### 3.4. Peruvian Attitudes towards HPV, HPV-Related Diseases, and HPV Vaccination

While 58% of American Hispanic women are aware that CC is an HPV-attributable disease, only 38% of urban Peruvian women are so aware [21]. Of note, Peruvian Quechua speaking women are more likely to be aware that HPV causes CC than are Peruvian Spanish speaking women or bilingual Peruvian women (42.4%, 29.9%, 36.2%) [21]. This disparity is also evident in awareness that HPV is a STI, with Quechua speaking women being most knowledgeable (86.7%, 80.7%, 84.9%) [21]. However, awareness that HPV vaccine prevents cancer and EGW was highest amongst bilingual Peruvian women (81%, 78.6%, and 87.8%) [21]. Most thought that HPV vaccine should be received after coitarche (61%, 76.7%, and 71.3%) [21].

Peruvian mothers are the primary vaccination decision maker in Peruvian households, with fathers having input [36,37]. Despite this, Peruvian parents are receptive to their daughters’ input about vaccination and allow their daughters to make their own vaccination choices [36,37]. Peruvian teachers and school principals are included in school-based vaccination program decision-making [36,37]. Peruvian children are aware of external stakeholders’ opinions about vaccinations [37]. Peruvians are aware that vaccination may be either primary or secondary prevention: for prophylaxis against disease or to ameliorate the course of disease [3,36,37]. There was very limited fear that HPV vaccination would provoke sexual disinhibition [36].

Before an HPV educational intervention 50% of participating CerviCusco clients were unaware that CC is preventable, and only 7% thought that the HPV vaccine effectively prevents HPV infection [33]. However, rural Peruvian parents make the decision to accept HPV vaccination faster than urban Peruvian parents who may review the situation with more people before arriving at a decision [36].

Peruvians have expressed concern about interactions between vaccinators and those receiving the vaccine, vaccinators’ training, needle reuse, post-expiration vaccine administration, and overall vaccine adverse events and safety [37]. Adverse events of previous vaccinations can negatively affect the decision to receive subsequent vaccinations [36]. Peruvian parents are particularly concerned that HPV vaccination could accelerate their daughters’ physiologic reproductive maturation [37]. Following 1990s directed sterilization programs some Peruvians have an underlying distrust of government sponsored health programs. Peruvian parents are concerned that HPV vaccination may be another form of sterilization [3,36,37]. However, other Peruvians maintain their trust in the Peruvian government to promote what is good for Peruvians [3]. Meanwhile, Peruvian children are concerned that HPV vaccination will give them cancer instead of preventing cancer [37]. Peruvians were most likely to refuse HPV vaccination due to “allergies”, the belief that the HPV vaccine is still experimental, or if dissuaded by others [46].

As Peruvian FSW may be brothel-based and use a given health clinic, promoting an HPV vaccination program for new Peruvian FSW was suggested [16]. Only 9.9% of Peruvian FSW were aware that CC is vaccine-preventable [16]. However, once aware of HPV vaccination to prevent CC 97.5% of Peruvian FSW and 90% of low-income urban-dwelling Peruvian women would accept HPV vaccination [16,19]. This is consistent with previous findings that knowledge of CC as an HPV-attributable disease increases the interest in getting the HPV vaccine (odds ratio [OR] = 2.39, *p* = 0.3) [21]. Most Peruvian FSW would pay US$27.70 for HPV vaccination, whereas 42% of low-income urban-dwelling Peruvian women would not pay for HPV vaccination [16,19]. In a subsequent study, 184 of 200 FSW completed a three-dose HPV vaccine trial, a 92% completion rate [34].

Among low-income urban-dwelling Peruvian women there are statistically significant associations between having at least a secondary education (OR 2.63, *p* > 0.000, 95% CI 1.71–4.02), parity (OR 2.49, *p* > 0.020, 95% CI 1.15–5.37), and recognition that HPV is causative of CC; between having health insurance (OR 1.82, *p* > 0.005, 95% CI 1.2–2.77), being interviewed in a health center (OR 1.54, *p* > 0.029, 95% CI 1.04–2.27) and awareness that the HPV vaccine prevents CC [19].

HPV prevalence in Peruvian men who have sex with men (MSM) is 77.1%, of which 47.3% are high risk HPV [41]. Peruvian transgender women (TW) may have an anogenital HPV prevalence of 95.6%, with a 47.8% HR HPV type prevalence [42]. Consistent with this, 77% of HIV negative MSM and TW have condom-less anal or oral sex [40]. EGW are traumatic for MSM, leading some to change from being the passive to the active partner, or to have sex in darkness [41]. Two-thirds of those with EGW would accept HPV vaccination for prophylaxis against HPV-attributable diseases [40]. Nonetheless, in a group of MSM and men who have sex with TW, some believed that the HPV vaccine controls, treats, or cures HPV-attributable disease [42]. Within this group, HPV vaccination was accepted for self-protection from HPV infection, to prevent transmission to others, to prevent EGW, and to be a role model, but recipients may not share their vaccination status with family or friends [42]. However, homophobia within Peruvian society and sexual health services provision, attitudinal lack of cancer prevention as a reason for receiving HPV vaccination, and the lack of routine male HPV vaccination, deter MSM and TW from requesting HPV vaccination [42].

Peruvian policy-makers have contradictory opinions regarding the importance of CC to the public and in terms of health care system priorities [3]. It is opined that if Peruvian CC statistics were perceived to be accurate, then CC would be of higher priority [3]. Participation of religious leadership in vaccination promotion programs may allay Peruvians distrust of vaccination [37]. Therefore, a successful vaccination program needs appropriate communication, well-educated vaccinators, and transparency in vaccine administration highlighting single use syringes and use of vaccine prior to expiration.

## 4. Discussion

### 4.1. Comparison of Attitudes towards HPV and HPV Vaccination across Andean Nations

Table 3 shows the range of aforementioned data. The lack of easily-accessible data on Bolivia and Ecuador is notable. Similarly, much more information is needed on Chile. Given differences in question phrasing or reporting, it is difficult to make direct comparisons across, and within, nations for which data is available. Nonetheless, rural to urban, lower to higher socioeconomic group, gender, and linguistic differences clearly exist within Colombia and Peru. Healthcare policy-making level differences compound the aforementioned differences across Colombia and Peru.

### 4.2. Means for Changing Attitudes towards HPV Vaccination

Increased knowledge of HPV, HPV-attributable diseases, and prophylaxis thereof requires education. While FSW in Peru can receive sexual health education through the special clinic from which health cards are received, MSM and TW do not access the public system in the same manner as FSW [40]. This may have contributed to an inverse knowledge disparity where MSM and TW with tertiary or more education are significantly less aware of HPV vaccination than are MSM and TW with secondary level or less education (*p* = 0.04) [40]. Parents, MSM, and TW should be educated that HPV vaccination has not been associated with either post vaccination sexual disinhibition (risk compensation) or increased post vaccination STI rates [42]. Governments should expand HPV vaccination programs to include adolescent boys, MSM, and TW [42]. Similarly, indigenous populations in the Andean region may have reduced access to health programs and an earlier coitarche need targeted HPV education programs [24]. Educational programs should target incorrect beliefs, such as that HPV vaccine should be received after coitarche (61%, 76.7%, and 71.3%) [21].

Disease severity and prophylactic vaccine availability have been linked to increased vaccine acceptance [32]. Increasing HPV knowledge serves to increase HPV vaccine acceptability and openness to CC screening [32]. Therefore, more culturally- and linguistically-appropriate general HPV public education should occur for all social groups and all sexual orientations [32,33,40]. Educational videos presented in clinics may also be useful [21]. Public health education media campaigns, photo- and telenovellas, and radio advertisements should run in both Spanish and Quechua [33]. Preventive health and vaccinations in general, as well as health clinic use can be promoted by media campaigns as preventive health use is associated with increased HPV vaccination acceptance [20].

Education should include safe sex practices and the need to reduce the lifetime number of sexual partners: Only 31.1% of sampled Chilean adolescents used condoms; 77% of HIV-negative Peruvian MSM and TW have condom-less anal or oral sex; In Colombia, age-specific HPV-16, -18, -31, and -58 prevalence is associated with more lifetime sexual partners; multiple sexual partners is a CC risk factor [23,32,40]. Education programs should realize that rural-urban dichotomies vary from one nation to another: HPV prevalence in rural Bolivia can be <17%, but in rural Colombia >35%, and rural Peruvian parents decide quicker to accept HPV vaccination than do urban Peruvian parents [23,24,25,36]. Preventive health campaigns may highlight that hormonal contraception normally induces a hypoestrogenic state, removing a co-factor for HPV infection severity [23]. Acknowledgement of vaccinator training for safe vaccine administration and reassurance of expected minor adverse effects including injection site pain should be included in public education campaigns [37]. Peruvians’ awareness that vaccines may be preventive or therapeutic will be beneficial in the future should the therapeutic vaccines and adoptive T-cell therapies currently under investigation for cervical intraepithelial neoplasia 2/3 and CC treatment reach the market [44,45].

## 5. Conclusions

Qualitative research regarding attitudes to and knowledge of HPV, HPV-related diseases, and HPV vaccines appears to be lacking for Bolivia and Ecuador. Much more work and data are needed in the English language literature for Chile than for Colombia and Peru. New research studies will be most effective when planned in the context of the extant literature to allow a comparison with the literature. This research is needed to help formulate effective HPV vaccination programs in Bolivia and Ecuador, and to address any HPV vaccination issues that may be specific to the Andean region. Colombian data show that most knowledge of HPV and HPV vaccination derives from media campaigns, followed by educational establishments [11]. Therefore, health care organizations need to increase efforts to correctly inform unvaccinated patients about HPV and HPV vaccination at every encounter. Similarly, clinic- and school-based HPV vaccination promotion efforts could be increased in quantity and effectiveness. Implementation of HPV vaccination programs may be advanced if locally- and nationally-prominent persons, including politicians and their partners become advocates for HPV vaccination programs [39].

## Figures and Tables

**Table 1:** Andean region cervical cancer (CC) incidence, mortality, screening, and socio-economic correlates.

	Bolivia	Chile	Colombia	Ecuador	Peru
2012 New CC cases	2029	1441	4661	2094	4636
2012 Standardized CC incidence per 100,000 women	47.7	2.8	18.7	29.0	32.7
2012 CC deaths	845	734	1986	1026	1715
2012 Standardized CC mortality per 100,000 women	21	6.0	8.0	14.0	12.0
Life expectancy (years)	69	82	78	79	77
2012 Potential years of life lost due to CC	185,108	19,818	45,678	24,624	37,730
Cytological cover	12.00	60.00	79.00	64.30	51.30
Estimated mortality under five years old per 1000	67.6	9.3	30.9	28.2	49.5
Per capita total health expenditure at International dollar rate	176	707	522	220	233
Proportion of population with access to improved sanitation	45	92	86	72	62
% of women screened	-	51.4	67	31.3	22.7
% false-negative Pap tests		<5			27
% abnormal Pap test follow-up	59	>90			25–34

Data extracted from [1,2].

**Table 2:** Selected reference summation.

**Epidemiology**		
**Reference**	**Nation(s)**	**Primary Results**
[1]	-	Epidemiology of cervical cancer (CC) in Latin America using GLOBOCAN data from 2000, 2008, and 2012 is summarized in Table 1.
[2]	Latin America	GLOBOCAN 2008 CC data with 2000–2008 demographic, socioeconomic, and public health correlates.
[7]	Colombia	Human papilloma virus (HPV)-16 and -18 are present in almost 20% cases of HNSCC.
[8]	-	HPV may be less involved than previously believed in HNSCC other than oropharyngeal cancer. Gender, global location, and cancer site affect HPV contribution to HNSCC.
[9]	-	Globally, HPV may be more prevalent in high grade squamous intraepithelial lesions of the penis than in invasive penile cancer.
[12]	Colombia	HPV-6, -11, and -16 were found in 69%, 27.1%, and 7.8% of HPV positive recurrent respiratory papillomatosis. Quadrivalent HPV vaccine (4vHPV) would be a more effective vaccine than bivalent HPV vaccine (2vHPV).
[13]	Peru	In Lima, HPV prevalence in female sex workers (FSW) ranges from 50.6 to 66.8%.
[22]	Colombia	HPV-31, -33, -45, and -58 may be more prevalent than HPV-18 in CC. Colombia would benefit from an HPV vaccine other than 2vHPV or 4vHPV.
[23]	Colombia	Age-specific seroprevalence of HPV-16, -18, -31, and -58 in women of a rural town was associated with a greater number of lifetime sexual partners (OR = 3.05, 95% confidence interval [CI] 1.26–7.37), and having >2 regular sexual partners (odds ratio [OR] 3, 95% CI 1.21–7.45). In women >45 years old, oral contraceptive use and tobacco smoking were associated with reduced HPV prevalence.
[24]	Colombia	Geographic region, ethnicity, parity, and lifetime number of sexual partners affect HPV infection.
[25]	Bolivia	HPV prevalence in 4 Amazonian villages ranged from 0 to 16.6%, related to enetic milieu or lifestyle factors limiting HPV exposure or effect.
[26]	Peru	In Lima, FSW oral HPV prevalence (7.6%) is associated with cervical HPV prevalence (71.4% if oral HPV positive).
[27]	Chile	From 2001 to 2005 female high-risk (HR) HPV infection rose by 43%. Dual peak HR HPV prevalence in women <20 years old and 45–55 years old. HR HPV infections in women <30 years old cleared in the five-year interval; 4.7% were HR HPV positive in 2001 and 2005.
[28]	Chile	From 2014 to 2015 in Santiago HPV prevalance in CC screening was 11.1%, of which 9.7% were high risk. HPV-66 (1.4%), -51 (1.2%), and -59 (1.2%), were more prevalent than HPV-18.
[29]	Chile	From 2012 to 2016 in Santiago, pap tests had a 12% HPV prevalence. Rates varied between primary care and referral centers.
[30]	Colombia, Finland	Coitarche within three years of menarche (12.4 and 16 years) increases cytologic abnormalities (OR 1.65, 95% CI 1.02–2.68, *p* = 0.04 and cervical intraepithelial neoplasia (CIN)2-3/adenocarcinoma in situ (AIS) (OR 3.56, 95% CI 1.02–12.47, *p* = 0.05).
**Knowledge and Knowledge Gaps**		
**Reference**	**Nation(s)**	**Primary Results**
[5]	Peru	The Piura region school-based HPV vaccination project required extensive vaccinator training, coordination with schools and education authories, sensitization of parents, adolescent daughters, teachers, and the community. Isolated, small schools were difficult and expensive (time and resources) to reach. Three-dose HPV vaccine administration tracking for each recipient was difficult.
[11]	Colombia	Men and women with external genital warts (EGW) had little HPV knowledge. EGW adversely affected women’s self esteem more than men’s self esteem (90.3% versus 60.4%, *p* < 0.001). EGW adversely affected women’s sexual life more than men’s (83% versus 66%, *p* = 0.05).
[16]	Peru	FSW in Lima have little HPV, HPV vaccine, and CC knowledge.
[31]	Chile	Chilean adolescents were aware that multiple sexual partners is a CC risk factor (70.8%) and a HPV infection risk factor (78.3%). While 68.2% were aware that unprotected sex facilitates HPV transmission, only 31.1% used condoms.
[32]	Colombia	Women attending clinics in Medellín were aware of CC screening (76.3%), but only 7.8% had HPV knowledge. Education, marital status, household income, and insurance affected CC screening knowledge. Education, age, and household income affected HPV knowledge.
[33]	Peru	Forty-six participant, mixed method, descriptive case study of CerviCusco programs. Explored HPV knowledge and CC attitudes and beliefs. Half of participants were unaware that CC is preventable.
[34]	Peru	FSW participation in a HPV vaccine trial was associated with increased HPV and HPV-related disease knowledge, increased awareness of HPV prevention strategies, and with a reduction in new and total clients, *p* = 0.001.
**Finance and Cost**		
**Reference**	**Nation(s)**	**Primary Results**
[4]	Peru	2vHPV vaccination of preadolescent girls with an 82% three-dose completion and 10% coverage CC screening of adult women would reduce the lifetime risk of CC by 58% at a cost of less than US$500 per year of life saved. Annual program cost ranges from US$5 million at US$5/dose to US$16 million at US$20/dose.
[17]	Chile	Parents of teenage daughters in Santiago were willing to pay US$758 for HPV vaccination series. But, 25% of parents would not pay for HPV vaccine. However, at half the price, 96% of parents would pay for HPV vaccine. Household income and size affect willingness to pay.
[18]	Chile	Double blinded format contingent valuation ascertained that parents of teenage daughters were willing to pay US$252.71 for HPV vaccination series in Maule region. Cost per dose and number of daughters lower the amount parents will pay. Household income raises the amount parents will pay. Shared funding between government and parents increases access of teenage daughters to HPV vaccination.
[19]	Peru	In Puente Piedra, Los Olivos, and Comas, Lima, although 59–71% of low-income 25–65 year old women were unaware of HPV, HPV vaccine, and CC, 90% would accept HPV vaccination, and 58% were willing to pay “something” for HPV vaccination.
**Policy**		
**Reference**	**Nation(s)**	**Primary Results**
[35]	Peru	A mother/daughter screen, treat, and vaccinate program around Iquitos, in the Peruvian jungle, achieved an 88% two-dose and a 65% three-dose 4vHPV vaccination rate.
[36]	Peru	If enough credible sources, including health workers and teachers promote HPV vaccine, parents will accept HPV vaccine. Health promotion, CC prevention, and trust in vaccines drive acceptance. Accessibility via free, school-based administration, and media campaigns motivate parental acceptance of HPV vaccination. Problems with the health system drive HPV vaccine refusal.
**Vaccination Barriers**		
**Reference**	**Nation(s)**	**Primary Results**
[20]	Colombia	HPV vaccination was not associated with sexual risk behavior young women in Bogotá. HPV vaccination was associated with other preventive health measures: Routine contraception and condom use, and CC screening. HPV vaccinated women percieved their risks of CC, EGW, and HPV infection to be less than did unvaccinated women.
[21]	Peru, United States	Spanish-speaking Peruvian women were most embarrassed and afraid to have pap tests (OR = 17.25, 14.43 respectively), less likely to know that the HPV vaccine is safe and effective (OR = 0.11), and less likely to know that HPV causes CC (OR = 0.03).
[37]	Peru	Adolescents’ mothers are vaccination decision-makers. Girls can choose for themselves. Teachers and principals are involved in school-based vaccination program decision-making. The Peruvian government has a role in determining what vaccines are available. Peruvian administrators, health care workers, and school staff have greater awareness of CC than do adolescents and their parents, but all had limited knowledge that CC is a HPV-related disease. Awareness that vaccines may be preventive or curative. Peruvians have concerns about vaccinators’ effective interactions with female HPV vaccine recipients and vaccination administration safety practices. Fear of vaccine-induced sterilization due to coercive sterilizations in the 1990s. Fear of vaccine induced precocious puberty and experimental vaccines.
[38]	Colombia	In Arauca, Bogotá, Cartagena, and Manizales parents of daughters younger than 12 years old were resistant to HPV vaccination to prevent sexually-transmitted infection (STI), and were concerned of sexual disinhibition, but would accept HPV vaccination to prevent CC. Parents of higher socioeconomic status were less accepting of HPV vaccination.
[39]	Colombia	Qualitative study. Interviews with HPV vaccine program decision-makers 1–2 years after introduction of 2vHPV and 4vHPV. Interest in CC and EGW prevention. Need for information sources other than pharmaceutical representatives, to reduce HPV vaccine cost. Lack of resources to vaccinate, screen, and treat. Ethics of economic inequity.
[40]	Peru	An HPV-related knowledge gap exists among men who have sex with men (MSM) and transwomen in Lima, that may be due to sex work stigmatization, STI underreporting, and lack of access to HPV vaccine.
[41]	Peru	MSM and transgender people in Lima had limited HPV knowledge, but were aware that EGW are a STI. EGW were stigmatizing, negatively affecting access to care, which was less used when EGW could not be effectively treated.
[42]	Peru	Focus group and individual interviews with 36 MSM and transgender women in Lima found mostly positive attitudes towards HPV vaccination, but concern for being stigmatized if positive HPV vaccination status was disclosed.
**Vaccination Efficacy**		
**Reference**	**Nation(s)**	**Primary Results**
[6]	-	The nonavalent HPV (9vHPV) vaccine generated antibody response to HPV 6,11, 16, and 18 is noninferior to the 4vHPV vaccine. 9vHPV vaccine is also prophylactic against infection and intraepithelial neoplasia in women from HPV-31, -33, -45, -52, and -58.
[14]	Colombia, Peru	4vHPV vaccine had 92.8% and 100% efficacy in preventing CIN and EGW, respectively, in Latin American women.
[43]	-	Phase 2b trial of VGX-3100 therapeutic synthetic DNA vaccine targeting HPV 16 and 18 E6 and E7 proteins for CIN 2/3 achieved 18.2 percentage point improved regression than placebo, *p* = 0.034.
[44]	-	Thirty percent histologic regression from CIN 2/3 to CIN 1 at 15 weeks following initial vaccination dose, in patients who received three doses of pNGVL4a-CRT-E7 (detox) vaccination.
[45]	-	Complete and partial regression of metastatic CC occurred in two of nine and one of nine patients, respectively, who received a single adoptive T-cell therapy infusion comprised of tumor-infiltrating T cells with possible HPV E6 and E7 reactivity.
**Vaccination Safety**		
**Reference**	**Nation(s)**	**Primary Results**
[3]	Peru	Policymakers and parents support HPV vaccine introduction, but communities have vaccination safety and quality concerns. Vaccines are seen as preventive not curative. A few parents were concerned about vaccine-related promiscuity and precocious puberty.
[46]	Peru	Piura region school-based HPV vaccination achieved a 82.6% three-dose completion rate. HPV vaccination was accepted for CC prevention and disease prevention due to positive beliefs concerning vaccination and free vaccination provision. HPV vaccination refusal was due to fear of experimentation, being dissuaded against HPV vaccination, and “allergies”. School absenteeism resulted in non-vaccination.

2vHPV, bivalent human papilloma virus vaccine; 4vHPV, quadrivalent human papilloma virus vaccine; 9vHPV, nonavalent human papilloma virus vaccine; AIS, adenocarcinoma in situ; CC, cervical cancer; CI, confidence interval; CIN, cervical intraepithelial neoplasia; EGW, external genital warts; FSW, Female sex workers; HR, high risk oncogenic; HPV, human papilloma virus; MSM, men who have sex with men; OR, odds ratio; HNSCC, squamous cell carcinoma of head and neck; STI, sexually transmitted infection; US, United States of America.

**Table 3:** Comparison of attitudes to and awareness of HPV and HPV vaccination across Andean Nations.

	Chile	Colombia			Peru
Aware that cervical cancer risk increases with more sexual partners	70.8% of adolescents				
Aware of HPV		44–69%. Higher with higher socioeconomic class			
Aware that HPV causes CC					Quechua women—42.4%; Bilingual women—36.2%; Spanish speaking women—29.9%; Urban women—38%
Aware of HPV vaccination		25–59%			
Aware that HPV vaccination prevents CC and external genital warts					Quechua women—81%; Bilingual women—87.8%; Spanish speaking women—78.6%
Aware that HPV is transmitted by unprotected sex (HPV is a sexually transmitted infection (STI))	68.2% of adolescents				Quechua women—86.7%; Bilingual women—84.9%; Spanish speaking women—80.7%
Normally use condoms	31.1% of adolescents				28% of HIV negative MSM and TW use condoms for anal or oral sex
Best time to receive HPV vaccination					After coitarche Quechua women—61%; Bilingual women—76.7%; Spanish speaking women—71.3%
Parents approve of HPV vaccinations for their daughters to prevent STI		Resistant			
Fear of post-vaccination sexual disinhibition		Concern does not override choice to prevent CC			Limited
Parents approve of HPV vaccinations for their daughters to prevent CC		Higher socioeconomic class parents are less supportive than lower socioeconomic class parents			Joint decision between parents, children, teachers, and school principles
Parental HPV vaccination acceptance decision					Rural parents are quicker to accept than urban parents
Actual HPV non-vaccination rate and reason		30%—lack of information			MSM
		28.9%—access barriers			Lack of a male HPV vaccination program
		21%—unknown			Non-realization of cancer prevention need
		20%—neglect			Societal and healthcare provision homophobia
External genital warts negative effect			Women	Men	MSMTraumatic, become the passive partner, start to have sex in darkness.
		Self-esteem	90%	62%	
		Sexuality	77%	46%	
External genital warts positive effect					Prompts MSM HPV vaccine acceptance
HPV knowledge sources		Media—82.25%; Social—72.4%; Educational—77.8%			
HPV vaccination knowledge sources		Media—59.8%; Social—44.8%; Educational—50%			
HPV vaccination concerns					Covert reproductive sterilization
					Covert cancer dissemination
					Experimental, unsafe vaccine
					Fear from previous vaccination adverse events
					Post-expiration vaccine administration
					Vaccine initiated reproductive development
					Vaccinator training inadequacy, non-sterile vaccination

Data extracted from references [3,11,16,17,18,19,20,21,30,31,32,33,34,36,37,38,39,40,41,42,46]. CC, cervical cancer; FSW, Female sex workers; HIV, human immunodeficiency virus; HPV, human papilloma virus; MSM, men who have sex with men; STI, sexually transmitted infection.
